# Can Chemoradiotherapy Expand Immune Checkpoint Blockade Responsiveness in Mismatch Repair‐Proficient Rectal Cancer?

**DOI:** 10.1002/ags3.70281

**Published:** 2026-09-16

**Authors:** Kimihiro Yamashita, Takeru Matsuda, Junko Mukohyama, Ryohei Sasaki, Yoshihiro Kakeji

**Affiliations:** ^1^ Division of Biophysics, Department of Health Sciences Kobe University Graduate School of Medicine Kobe Hyogo Japan; ^2^ Department of Surgery, Division of Gastrointestinal Surgery Kobe University Graduate School of Medicine Kobe Hyogo Japan; ^3^ Department of Surgery, the Institute of Medical Science The University of Tokyo Tokyo Japan; ^4^ Division of Radiation Oncology Kobe University Graduate School of Medicine Kobe Hyogo Japan

**Keywords:** chemoradiotherapy, immune checkpoint blockade, mismatch repair‐proficient, pathological complete response, rectal cancer

## Abstract

Immune checkpoint blockade (ICB) has transformed the treatment of mismatch repair‐deficient (dMMR) colorectal cancer but produces no meaningful benefit in advanced mismatch repair‐proficient (pMMR) disease. In the neoadjuvant setting, however, pathological responses have been reported in a subset of pMMR tumors, suggesting that clinical context modifies apparent ICB sensitivity. In pMMR rectal cancer, adding ICB to chemoradiotherapy (CRT) has been associated with pathological complete response (pCR) rates of 30% or more in several trials, higher than expected with CRT alone. These data are clinically provocative, particularly because sustained clinical complete response (cCR) may support watch‐and‐wait, but pCR and cCR remain early endpoints; long‐term disease control and organ‐preservation safety remain insufficiently defined. Whether CRT induces ICB responsiveness remains unresolved. The increase in pCR with sequential PD‐1 blockade after CRT provides a clinical signal for testing this possibility, but pCR cannot distinguish CRT‐induced ICB responsiveness from the simple additive effects of CRT and ICB. Pretreatment T‐cell‐inflamed features are candidate enrichment markers, but none is validated for patient selection. Priorities are therefore to compare biomarkers associated with ICB responsiveness before and after CRT within the same patients and prospectively validate selection assays. Long‐term follow‐up must determine whether treatment‐induced increases in pCR are accompanied by better disease control and whether organ preservation after sustained cCR is safe.

## Abstract 2

Adding immune checkpoint blockade (ICB) to neoadjuvant chemoradiotherapy (CRT) has been associated with pathological complete response (pCR) rates of 30% or more in mismatch repair‐proficient rectal cancer, higher than expected with CRT alone. Whether CRT induces ICB responsiveness or the higher pCR simply reflects the additive effects of CRT and ICB remains unresolved, and pCR remains an early endpoint. This review outlines the clinical evidence and two prospective biomarker priorities: within‐patient comparisons before and after CRT to test the induction hypothesis, and prospective validation of assays for patient selection.

## 1 Introduction

The management of locally advanced rectal cancer has been transformed by neoadjuvant chemoradiotherapy (CRT) or short‐course radiotherapy prior to surgery, progressively improving downstaging and enabling organ preservation [1]. Immune checkpoint blockade (ICB) has since emerged as a further therapeutic option, but one whose benefits are distributed in a strikingly asymmetric manner across mismatch repair (MMR) status.

In mismatch repair‐deficient (dMMR) colorectal cancer (CRC), ICB produces durable responses across disease stages: median overall survival reaches 77.5 months in the advanced setting [2, 3], and neoadjuvant ICB achieves pCR rates of 60%–68% [4, 5], with a subset of patients achieving sustained clinical complete response without surgery (Table 1) [8, 9]. These results have redefined the treatment of dMMR disease. In contrast, mismatch repair‐proficient (pMMR) CRC, representing approximately 95% of metastatic CRC, shows no meaningful benefit from ICB in advanced disease [6, 7] and pMMR status has been widely regarded as a surrogate marker of ICB resistance.

**TABLE 1:** Comparison of dMMR and pMMR colorectal cancer in the context of immune checkpoint blockade.

	dMMR (MSI‐H)	pMMR (MSS)
Frequency	∼15% (Stage II/III); ∼5% (Stage IV)	∼85% (all‐stage); ∼95% (metastatic)
TMB	High (abundant neoantigens)	Low (few neoantigens)
Immune microenvironment	Inflamed (“hot”)	Non‐inflamed (“cold”)
ICB in advanced disease	Highly effective (median OS 77.5 months)	Ineffective
Neoadjuvant ICB	pCR 68% (NICHE‐2)	pCR ∼10% (expanded pMMR NICHE cohort)

*Note:* References: advanced dMMR [2, 3]; advanced pMMR [6, 7]; neoadjuvant dMMR [4, 8, 9]; neoadjuvant pMMR [5, 10]. Clinical complete responses (watch‐and‐wait, no surgery) are not tabulated: dostarlimab achieved clinical complete response in all 49 patients with dMMR rectal cancer who completed treatment [9] and one patient in the expanded pMMR NICHE cohort had an ongoing cCR [10].

Abbreviations: cCR, clinical complete response; dMMR, mismatch repair‐deficient; ICB, immune checkpoint blockade; MSI‐H, microsatellite instability‐high; MSS, microsatellite stable; OS, overall survival; pCR, pathological complete response; pMMR, mismatch repair‐proficient; TMB, tumor mutational burden.

Yet this resistance may not be absolute. In pMMR rectal cancer, adding ICB to neoadjuvant CRT has been associated with higher pCR in selected Phase II trials, but the current evidence establishes clinical activity of specific regimens rather than proving that CRT induces ICB responsiveness. Throughout, we use pCR as an early objective endpoint, not as a validated surrogate for survival or durable organ preservation: although patients who achieve pCR have better long‐term outcomes [11], treatment‐induced increases in pCR have not been shown to improve 5‐year survival [12]. High response rates make organ preservation conceivable if a sustained cCR permits watch‐and‐wait rather than radical surgery. This review examines the clinical evidence for CRT plus ICB in pMMR rectal cancer and outlines prospective biomarker strategies to test whether CRT induces ICB responsiveness and to guide patient selection.

## 2 The pMMR Paradox and Neoadjuvant CRT

In the NICHE study of non‐metastatic colon cancer, neoadjuvant nivolumab plus ipilimumab produced responses in pMMR tumors: an expanded cohort showed responses in 8 of 31 (26%), including major pathological responses in 6 of 30 (20%) and pathological complete response (pCR) in 3 of 30 (10%); one of these responders had an ongoing clinical complete response (cCR) without surgery [5, 10]. This result showed that pMMR tumors, although resistant to ICB in advanced disease, can still respond in the neoadjuvant setting. Comparable data in pMMR rectal cancer do not exist, because neoadjuvant therapy there is built around CRT rather than ICB alone; NICHE therefore remains the principal reference.

In the metastatic setting, RT combined with ICB has failed to produce meaningful responses in pMMR CRC [13, 14]. By contrast, in neoadjuvant pMMR rectal cancer, the single‐arm VOLTAGE and NECTAR trials reported pCR rates of 30%–40% with ICB‐containing regimens based on standard fluoropyrimidine CRT, while randomized POLARSTAR showed that sequential PD‐1 blockade significantly increased pCR versus CRT alone (Table 2) [15, 16, 17]. This clinical signal is early evidence, not proof of durable benefit. Moreover, pCR at surgery is not equivalent to the sustained cCR required for watch‐and‐wait, and organ‐preservation safety further depends on local regrowth and salvage success: in a meta‐analysis of watch‐and‐wait series, ultimate local failure was 5% overall but 24% with regrowth [21].

**TABLE 2:** Clinical trials of neoadjuvant chemoradiotherapy (CRT) combined with immune checkpoint blockade (ICB) in pMMR rectal cancer. Trials are grouped by chemotherapy backbone to keep pCR comparable; Oxaliplatin‐intensified and biomarker‐selected trials are listed separately because their pCR is not directly comparable to standard‐backbone trials.

Trial	Design	ICB and timing	Chemo backbone	pCR
*Standard CRT backbone (fluoropyrimidine‐based)*				
VOLTAGE [15]	Phase I/II, single‐arm (MSS *n* = 37)	Nivolumab, sequential (after CRT)	Standard	30% (11/37)
NECTAR [16]	Phase II, single‐arm (*n* = 50)	Tislelizumab, concurrent	Standard	40% (20/50)
POLARSTAR [17]	Phase II RCT, 3‐arm (*n* = 186; MMR‐unselected; 4 dMMR confirmed)	PD‐1, concurrent vs sequential (timing isolated)	Standard	Control 14.0%; concurrent 27.1% (NS); sequential 32.7% (RR 2.332, *p* = 0.019)
CHINOREC [18]	Phase II RCT (*n* = 80; primary endpoint safety)	Dual ICB (ipilimumab + nivolumab), concurrent	Standard	CR (clinical/pathological)^a^ : CRT alone 30% vs. CRT + dual ICB 22% (*p* = 0.44)
*Oxaliplatin‐intensified concurrent chemo*				
Sintilimab RCT (Xiao) [19]	Phase II RCT (*n* = 134)	Sintilimab, concurrent	Intensified (CAPOX‐CRT)	Total CR 26.9% (CRT alone) vs. 44.8% (+ sintilimab); *p* = 0.031^b^
*Intensified + biomarker‐selected*				
SILAR [20]	Phase II, single‐arm (*n* = 46)	Sintilimab, concurrent	Intensified (mFOLFOX6‐CRT); Immunoscore‐selected^c^	65.2% (30/46)

*Note:* ICB timing: concurrent, ICB during CRT; sequential, ICB after CRT completion. Chemo backbone: standard, fluoropyrimidine‐based (e.g., capecitabine); intensified, oxaliplatin‐containing (CAPOX [capecitabine plus oxaliplatin] or mFOLFOX6 [modified leucovorin, fluorouracil, and oxaliplatin]). Oxaliplatin intensification raises pCR independently of ICB, so intensified‐backbone pCR is not directly comparable to standard‐backbone trials. Total neoadjuvant therapy (TNT)‐based regimens are discussed separately in the text and are not included here.

Abbreviations: CD, cluster of differentiation; CR, complete response (pCR + clinical complete response); CRT, chemoradiotherapy; dMMR, mismatch repair‐deficient; ICB, immune checkpoint blockade; MMR, mismatch repair; MSS, microsatellite stable; NS, not significant; pCR, pathological complete response; PD‐1, programmed cell death protein 1; pMMR, mismatch repair‐proficient; RCT, randomized controlled trial; RR, risk ratio.

^a^ CHINOREC reported complete response (clinical or pathological); the primary endpoint was safety. Not directly comparable to pCR.

^b^ Primary endpoint was total CR (pCR + clinical complete response).

^c^ Enrolled only intermediate/high Immunoscore (CD3^+^ and CD8^+^ T‐cell density) patients; the high pCR reflects patient selection and is not directly comparable to unselected trials.

Three randomized Phase II trials clarify where this clinical signal emerges. The most directly informative randomized timing comparison comes from POLARSTAR, which tested single‐agent PD‐1 in a predominantly pMMR population: sequential PD‐1 after CRT reached significance (pCR 14.0% with CRT alone vs. 32.7%; RR 2.332, *p* = 0.019), whereas the concurrent arm reached 27.1%, a numerically smaller increase that did not reach significance [17]. CHINOREC showed that adding dual ICB (ipilimumab and nivolumab) concurrently with CRT did not improve complete response (clinical or pathological; 30% with CRT alone vs. 22%; *p* = 0.44) [18]. In the sintilimab trial, single‐agent PD‐1 added to intensified CAPOX‐CRT increased the composite pCR or cCR rate from 26.9% to 44.8% (*p* = 0.031) [19]. Together, these trials show that the benefit depends on how ICB is combined with CRT.

Selection by a pre‐treatment immune marker may also enrich for responders. In the single‐arm SILAR trial, choosing patients with intermediate or high pre‐treatment Immunoscore (CD3^+^ and CD8^+^ T‐cell density) was associated with a pCR rate of 65.2% under an intensified CRT plus sintilimab regimen, supporting the feasibility of immune‐marker‐based enrichment, although without a low‐Immunoscore comparator it cannot establish Immunoscore as a validated predictive biomarker (Table 2) [20].

## 3 Immunological Basis of ICB Responsiveness in pMMR CRC

Why might ICB succeed before surgery in some pMMR tumors yet fail once the disease has advanced? Current evidence suggests that this paradox is unlikely to be explained by a single mechanism, and that two levels should be distinguished. The first concerns the disease setting and whether conditions that support ICB responsiveness are preserved. The second concerns variation within a setting, where the antitumor immune state present before treatment separates tumors that respond from those that do not.

**FIGURE 1:**
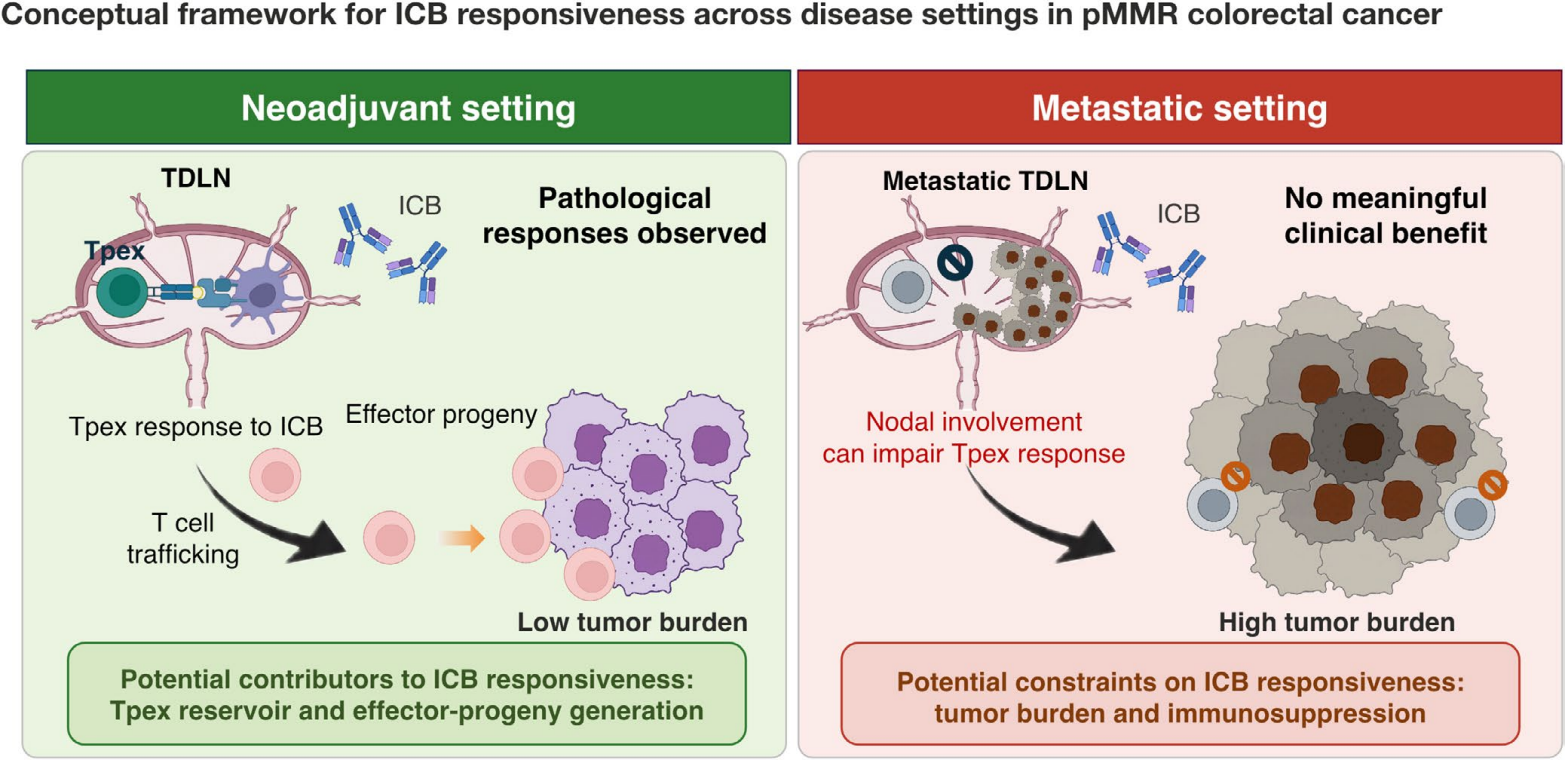
Conceptual framework for ICB responsiveness across disease settings in pMMR colorectal cancer. In the neoadjuvant setting, the Tpex reservoir and generation of effector progeny are shown as potential contributors to ICB responsiveness. In the metastatic setting, nodal involvement can impair the Tpex response, while tumor burden and immunosuppression are shown as potential constraints on ICB responsiveness. This framework is extrapolated from broader ICB biology [22, 23, 24, 25, 26, 27] and has not been directly validated in pMMR rectal cancer. The figure depicts these setting‐level conditions only; the pre‐existing antitumor immune state, the second level discussed in the text, is not shown. Abbreviations: ICB, immune checkpoint blockade; pMMR, mismatch repair‐proficient; TDLN, tumor‐draining lymph node; Tpex, progenitor‐exhausted CD8^+^ T cells.

First, conditions that support ICB responsiveness may be better preserved before surgery (Figure 1). Tumor‐draining lymph nodes (TDLNs) are increasingly recognized as important sites of the response to PD‐1/PD‐L1 blockade. TCF1 is expressed by naive and memory T cells and by progenitor‐exhausted CD8^+^ T cells (Tpex), and is lost on terminal differentiation [28]. Intratumoral Tpex sustain tumor control under ICB [22] and TDLNs harbor Tpex that expand after ICB and generate progeny trafficking to the tumor [23, 24]. In head and neck cancer, the Tpex response to ICB was retained in uninvolved nodes but impaired in metastatic nodes, suggesting that nodal involvement can disrupt this immune compartment [25]. Advanced disease also carries a larger tumor burden, deeper T‐cell exhaustion [26] and a more immunosuppressive microenvironment [27]. These conditions are inferred from broader ICB biology and have not been directly validated in pMMR rectal cancer.

Second, within such a setting, ICB amplifies antitumor immunity that is already present rather than creating it, so the immune state before treatment constrains what ICB can achieve. In the expanded pMMR NICHE cohort, response occurred despite uniformly low tumor mutational burden and tracked instead with the pre‐treatment immune state, including higher expression of TCF1, which is not specific to Tpex; a fibrotic, TGF‐β‐rich stroma marked many non‐responders [10]. This dissociation between mutation burden and response is a recurring theme across gastrointestinal cancers [29]. These features describe a pre‐existing immune competence that varied continuously rather than falling into discrete categories, an observation so far limited to this single cohort.

Taken together, these two levels offer a rationale for the paradox: the neoadjuvant setting preserves conditions under which ICB can act, while the pre‐existing immune state accounts for the variation that remains within that setting.

## 4 CRT and the Path to pCR

**FIGURE 2:**
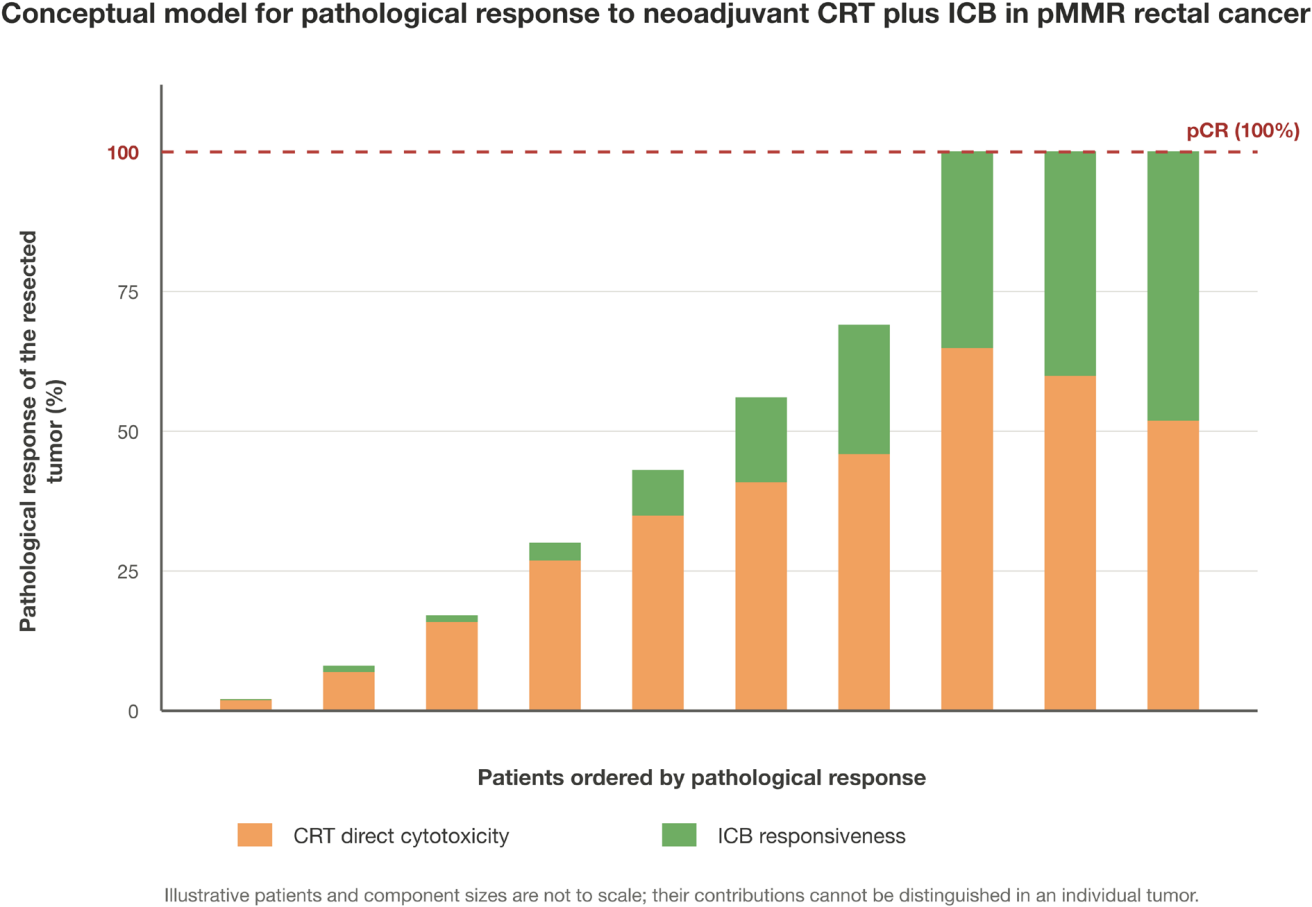
Conceptual model for pathological response to neoadjuvant CRT plus ICB in pMMR rectal cancer. Each bar represents one patient; its height is the pathological response of the resected tumor, with 100% corresponding to pathological complete response (pCR, dashed line). Each bar is conceptually divided into CRT direct cytotoxicity (orange), defined as tumor‐cell death independent of ICB, and ICB responsiveness (green), the component of response that requires ICB. At the population level, CRT alone can produce pCR in rectal cancer [17] and neoadjuvant ICB alone has also produced pCR in a minority of pMMR colon cancers [10]. At the individual level after CRT plus ICB, however, the observed response cannot be partitioned between these components. ICB responsiveness varies continuously across patients rather than separating them into responders and non‐responders; this continuum is extrapolated from neoadjuvant ICB in the pMMR NICHE colon cancer cohort [10]. Abbreviations: CRT, chemoradiotherapy; ICB, immune checkpoint blockade; pCR, pathological complete response; pMMR, mismatch repair‐proficient.

The pathological response to CRT plus ICB can be viewed as reflecting both CRT direct cytotoxicity and ICB responsiveness (Figure 2). Evidence from the pMMR NICHE colon cancer cohort suggests that ICB responsiveness varies continuously rather than falling into responder and non‐responder categories [10]. If this continuum extends to pMMR rectal cancer, shifting the distribution of pathological response could increase the proportion of patients reaching pCR.

At the regimen level, this could be pursued by strengthening the treatment backbone or refining how CRT and ICB are delivered. Treatment intensification ranges from an oxaliplatin‐containing CRT backbone, as in CAPOX‐CRT [19], to ICB‐containing TNT; early trials of the latter have reported pCR or composite pCR/cCR rates of approximately 40%–60% [30]. Potential refinements include radiotherapy dose and schedule, ICB timing, and TDLN coverage within the radiation field, although their optimal use remains uncertain.

CRT may promote immunogenic cell death and antigen presentation, providing a biological rationale for greater responsiveness to subsequent ICB [31, 32, 33]. POLARSTAR provides a clinical signal regarding timing: sequential PD‐1 blockade after CRT significantly increased pCR versus CRT alone, whereas the smaller numerical increase in the concurrent arm was not significant [17]. Together, this biological rationale and clinical signal support testing whether CRT can induce—and thereby expand—ICB responsiveness, rather than the higher pCR reflecting only the simple additive effects of CRT and ICB. Because pCR cannot distinguish CRT‐induced ICB responsiveness from simple additivity, larger prospective trials should compare biomarkers associated with ICB responsiveness before and after CRT within the same patients.

Because treatment intensification exposes all patients to additional therapy, biomarker‐based selection could help identify those most likely to benefit. The leading enrichment candidate is a pretreatment T‐cell‐inflamed phenotype, assessed by Immunoscore or CD3⁺/CD8⁺ density. Immunoscore was used prospectively in SILAR, which reported a high pCR rate [20], while T‐cell density was associated with response in earlier rectal CRT studies [34, 35]. Other signals include higher pretreatment TCF1 in responders and a TGF‐β‐rich stroma in nonresponders in the pMMR NICHE colon cohort [10] and an exploratory association between PD‐L1 combined positive score and sintilimab benefit in the randomized trial [19]. IFN‐γ and antigen‐presentation signatures remain plausible but untested candidates. None is yet a clinically validated selection assay. These candidates warrant deeper investigation using tumor samples available in the neoadjuvant setting, where their associations with pathological response can be assessed directly.

Most evidence concerns pretreatment biomarkers. If ICB is administered after CRT, assessment at the post‐CRT/pre‐ICB time point may provide a clinically relevant estimate of the likelihood of benefit from ICB at the point of treatment selection. Developing and prospectively validating an assay for this purpose is therefore the task ahead.

## 5 Conclusion

Resistance to ICB in pMMR rectal cancer is not absolute: selected neoadjuvant CRT plus ICB regimens have produced higher pCR rates than expected with CRT alone. These findings support testing whether CRT can induce—and thereby expand—ICB responsiveness, but pCR cannot distinguish this from simple additivity. Further regimen optimization should proceed together with prospective validation of biomarkers for patient selection. Within‐patient biomarker comparisons before and after CRT can separately evaluate the induction hypothesis. Long‐term follow‐up is needed to determine whether treatment‐induced increases in pCR are accompanied by better disease control and whether organ preservation after sustained cCR is safe.

## Author Contributions

**Junko Mukohyama:** writing – review and editing. **Kimihiro Yamashita:** conceptualization, investigation, visualization, project administration, funding acquisition, writing – original draft, writing – review and editing. **Takeru Matsuda:** funding acquisition, writing – review and editing. **Ryohei Sasaki:** writing – review and editing. **Yoshihiro Kakeji:** funding acquisition, writing – review and editing, supervision.

## Funding

This research was funded by JSPS KAKENHI Grant Number JP24K02519 (Yoshihiro Kakeji), JP24K11929 (Takeru Matsuda), and JP23K08171 (Kimihiro Yamashita).

## Ethics Statement

The authors have nothing to report.

## Conflicts of Interest

Junko Mukohyama received consulting fees from Nippon Kayaku Co. Ltd. Yoshihiro Kakeji received payment or honoraria for lectures, presentations, speakers bureaus, manuscript writing or educational events from MSD K.K. All other authors declare no competing interests.

## Data Availability

Data sharing not applicable to this article as no datasets were generated or analysed during the current study.
